# Patients with dementia with Lewy bodies display a signature alteration of their cognitive connectome

**DOI:** 10.1038/s41598-024-84946-4

**Published:** 2025-01-06

**Authors:** Roraima Yanez-Perez, Eloy Garcia-Cabello, Annegret Habich, Nira Cedres, Patricia Diaz-Galvan, Carla Abdelnour, Jon B. Toledo, José Barroso, Daniel Ferreira

**Affiliations:** 1 Division of Clinical Geriatrics, Centre for Alzheimer Research, Department of Neurobiology, Care Sciences, and Society, Karolinska Institutet, Stockholm, Sweden; 2 Department of Clinical Psychology, Psychobiology and Methodology, Faculty of Psychology, University of La Laguna, Canary Islands, Spain; 3 Department of Psychology, Faculty of Health Sciences, University Fernando Pessoa Canarias, Las Palmas, Spain; 4 University Hospital of Psychiatry and Psychotherapy, University of Bern, Bern, Switzerland; 5 Department of Psychology, Sensory Cognitive Interaction Laboratory (SCI-lab), Stockholm University, Stockholm, Sweden; 6 Department of Radiology, Mayo Clinic, Rochester, MN USA; 7 Servicio de Neurología y Neurofisiología Clínica, Instituto de Biomedicina de Sevilla, Unidad de Trastornos del Movimiento, Hospital Universitario Virgen del Rocío, CSIC/Universidad de Sevilla, Seville, Spain; 8 Department of Neurology and Neurological Sciences, Stanford University School of Medicine, Stanford, CA USA; 9 Stanley H. Appel Department of Neurology, Nantz National Alzheimer Center, Houston Methodist Hospital, Houston, TX USA; 10 Department of Neurobiology, Care Sciences and Society (NVS), Center for Alzheimer Research, Division of Clinical Geriatrics, NEO floor 7th, 141 83 Huddinge, SE Sweden

**Keywords:** Cognition, Neurodegenerative disease, Graph theory, Connectome, Cognitive ageing, Dementia, Neurodegenerative diseases

## Abstract

Cognition plays a central role in the diagnosis and characterization of dementia with Lewy bodies (DLB). However, the complex associations among cognitive deficits in different domains in DLB are largely unknown. To characterize these associations, we investigated and compared the cognitive connectome of DLB patients, healthy controls (HC), and Alzheimer’s disease patients (AD). We obtained data from the National Alzheimer’s Coordinating Center. We built cognitive connectomes for DLB (*n* = 104), HC (*n* = 3703), and AD (*n* = 1985) using correlations among 24 cognitive measures mapping multiple cognitive domains. Connectomes were compared using global and nodal graph measures of centrality, integration, and segregation. For global measures, DLB showed a higher global efficiency (integration) and lower transitivity (segregation) than HC and AD. For nodal measures, DLB showed higher global efficiency in most measures, higher participation (centrality) in free-recall memory, processing speed/attention, and executive measures, and lower local efficiency (segregation) than HC. Compared with AD, DLB showed lower nodal strength and local efficiency, especially in memory consolidation. The cognitive connectome of DLB shows a loss of segregation, leading to a loss of cognitive specialization. This study provides the data to advance the understanding of cognitive impairment and clinical phenotype in DLB, with implications for differential diagnosis.

**Supplementary Information:**

The online version contains supplementary material available at 10.1038/s41598-024-84946-4.

## Introduction

Dementia with Lewy bodies (DLB) is a common neurodegenerative dementia^1^. The essential criterion for diagnosing DLB is a progressive cognitive decline^2^. In addition, characterization of that cognitive decline plays an important role in the differential diagnosis of DLB^3^. The typical cognitive profile of DLB includes deficits in attention, executive functions, and visual abilities, while other domains such as memory can be involved at later stages of the disease^2^.

The traditional approach when investigating cognition in DLB is to focus on the performance of a particular cognitive measure using univariate statistical analysis and comparing DLB patients with healthy controls (HC) or other dementias that are relevant for the differential diagnosis of DLB, such as Alzheimer’s disease (AD) or Parkinson’s disease with dementia^4–6^. Although that univariate approach can provide information on the cognitive profile of DLB patients, it falls short when elucidating the complex associations between cognitive domains. This approach contrasts with how cognition is in fact assessed and interpreted in clinical settings, where the clinician considers all cognitive measures at once rather than conclude based on a single cognitive test. This issue highlights the necessity of using multivariate approaches to investigate cognition in DLB. This is also reinforced by a recent narrative review that suggested that the impairment in some cognitive domains in DLB is secondary to impairments in other central cognitive domains^7^. However, to our knowledge, no previous study has used a multivariate approach to assess associations between cognitive measures in DLB in comparison with controls or other relevant diagnostic groups. The recent study by Matar and colleagues^8^ used factor analysis on items from the Montreal Cognitive Assessment (MoCA)^9^, but the complex associations among cognitive domains are still poorly understood in DLB.

The “cognitive connectome”^10^ is a new concept and methodology that comprehensively represents the complex organization and associations among cognitive domains in a population. Using graph theory analysis on cognitive measures provides rich data on the centrality of specific cognitive measures and information about the integration and segregation of the cognitive connectome^11^. While the cognitive connectome has been investigated in normal aging^10,12^ and some clinical conditions such as epilepsy, acquired brain injury, vascular encephalopathy, Parkinson’s disease, mild cognitive impairment, and AD^11,13–18^, no study to date has investigated the cognitive connectome in DLB. Characterizing the cognitive connectome in DLB could have implications for advancing our understanding of the complex and heterogeneous clinical phenotype of DLB. Moreover, it could help improve the differential diagnosis of DLB by integrating data profiles instead of assessing each cognitive measure separately in an “univariate” manner. Such a connectome approach will better align with how clinicians assess and interpret cognitive data.

In this study, we introduced graph theory analysis on cognitive measures in patients with DLB to investigate their cognitive connectome. The first objective was to characterize the cognitive connectome in DLB through a comparison with HC. The second objective was to compare the cognitive connectome of DLB and AD patients, as the most common comparison in the differential diagnosis of DLB in the clinical setting. We hypothesized prominent alterations in the cognitive connectome of DLB patients in comparison with HC, particularly involving attention, executive, and visual domains. In contrast, we expected modest differences between the cognitive connectomes of DLB and AD patients, with main differences involving memory measures and possibly extending to other cognitive domains.

## Results

### Cohort characteristics

Table 1 Shows the main demographic and clinical characteristics of the groups. There were no statistically significant differences in age for DLB compared with HC and AD, while there were significantly more men in DLB than in both HC and AD, and DLB had more years of education than AD. DLB and AD groups did not differ in the CDR total score. DLB showed statistically significantly lower performance in all cognitive domains compared with HC. Further, DLB showed significantly lower performance in the processing speed/attention domain compared with AD; and higher performance in visual and verbal episodic memory, language, and orientation compared with AD.

**Table 1:** Key demographic and clinical characteristics.

	DLB (*N* = 104)	HC (*N* = 3703)	AD (*N* = 1985)	DLB vs. HC	DLB vs. AD	
	Mean (SD)	Mean (SD)	Mean (SD)	*p*-value	*p*-value	
Age, years min-max	71.9 (8.5) 45–88	72.2 (9.2) 45–101	73.7 (9.9) 45–103	0.7	0.1	
Sex, men (%)	86%	43%	47%	< 0.001	< 0.001	
Education, years	16.3 (3.1)	16.0 (2.9)	15.4 (3.0)	0.3	< 0.01	
CDR-total, median (interquartile range)	1 (0.5-1)	0 (0–0)	1 (0.5-1)	< 0.001	0.2	
Cognitive fluctuations, present (%)	60%	1%	4%	< 0.001	< 0.001	
Visual hallucinations, present (%)	43%	1%	4%	< 0.001	< 0.001	
Probable RBD, present (%)	65%	1%	3%	< 0.001	< 0.001	
Parkinsonian signs, present (%)	82%	3%	11%	< 0.001	< 0.001	
Cognitive domains						
Visuoconstructive functions	-1.8 (1.8)	0 (0.8)	-1.6 (1.9)	< 0.001	0.1	
Visual and verbal episodic memory	-1.5 (0.9)	0 (0.6)	-2.1 (0.9)	< 0.001	< 0.001	
Executive functions	-0.9 (0.8)	0 (0.5)	-0.9 (0.7)	< 0.001	0.2	
Processing speed/attention	-2.1 (1.6)	0 (0.7)	-1.7 (1.7)	< 0.001	< 0.01	
Language	-1 (1.1)	0 (0.8)	-1.5 (1.6)	< 0.001	< 0.001	
Orientation	-3.1 (4.2)	0 (1)	-6.9 (5.5)	< 0.001	< 0.001	

Mean (standard deviation) reported in the table, otherwise specified. Cognitive domains expressed as averaged z-scores. For CDR, there was missing data for 1 DLB, 411 HC and 16 AD. For cognitive fluctuations, there was missing data for 2 DLB, 1 HC, and 41 AD. For visual hallucinations, there was missing data for 1 HC and 3 AD. Probable RBD data was missing for 3 DLB, 7 HC, and 26 AD. Comparisons were established a priori for DLB vs. HC and DLB vs. AD. Abbreviations: CDR, Clinical Dementia Rating scale; RBD, rapid eye movement sleep behavior disorder. DLB: Dementia with Lewy bodies; AD: Alzheimer’s Disease; HC: healthy controls.

## Weighted correlations matrices

**Fig. 1:**
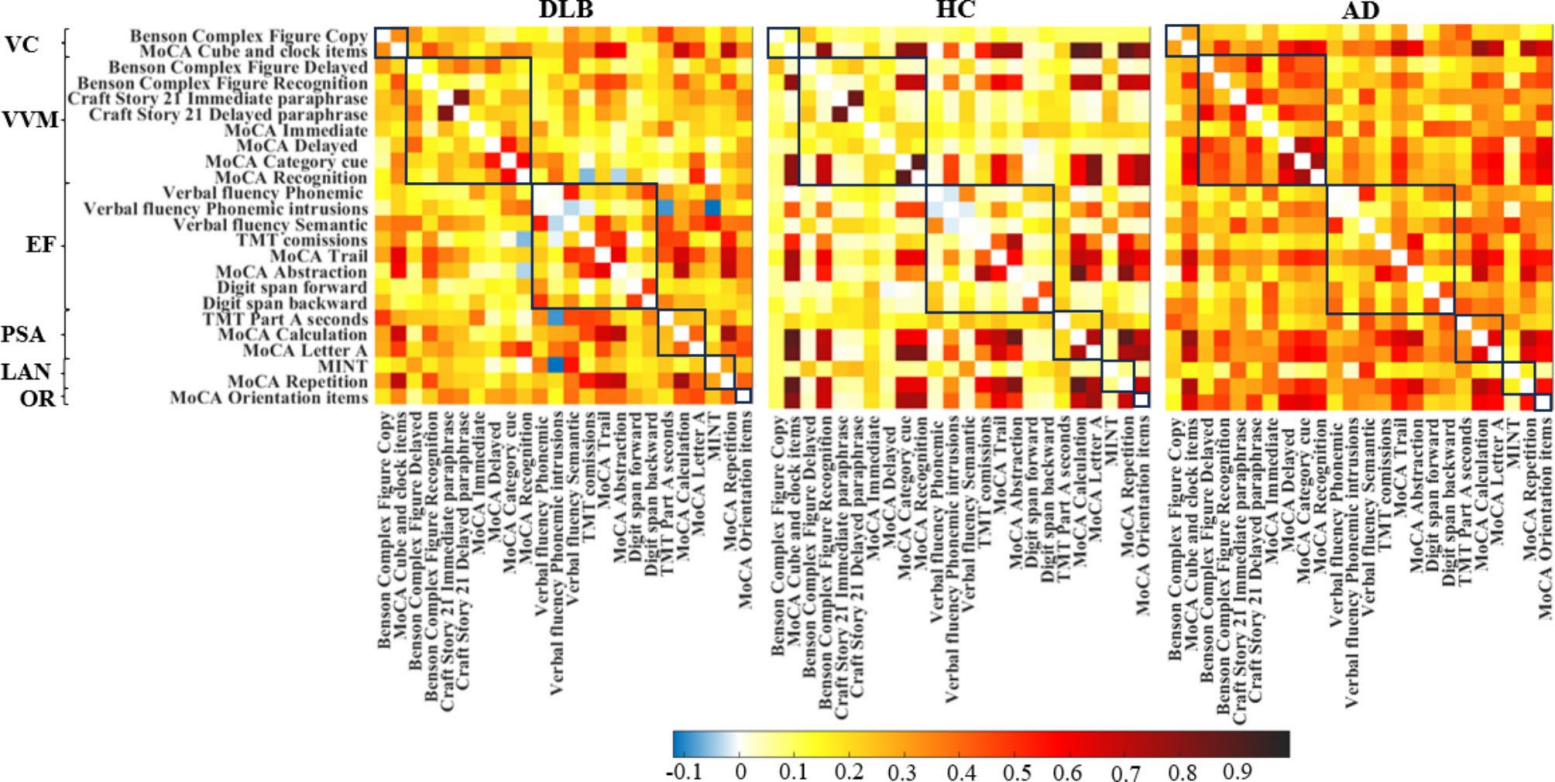
Correlation matrices of DLB, HC, and AD groups with cognitive measures grouped by cognitive domain. Negative correlations were observed only in the DLB and HC groups. The color bar shows the same correlation coefficients range for all three groups. Abbreviations: VC, visuoconstructive functions; VVM, visual and verbal episodic memory; EF, executive functions; PSA, processing speed/attention; LAN, language; OR, orientation; TMT: Trail Making Test; MINT: Multilingual Naming Test; MoCA: Montreal Cognitive Assessment.

Figure 1 shows the cognitive connectome of each study group. Visual inspection of the cognitive connectome in DLB showed uniformly weak correlations within and between the six cognitive domains (matrices sorted out by cognitive domains, with black boxes depicting domains to facilitate interpretation). In contrast, visual inspection of the cognitive connectome in HC showed coexisting strong and weak correlations within and between cognitive domains. Visual inspection of the cognitive connectome in AD suggested a similar pattern to that observed in DLB, with a predominance of weak to moderate correlations within and between cognitive domains.

## Global graph measures

**Fig. 2:**
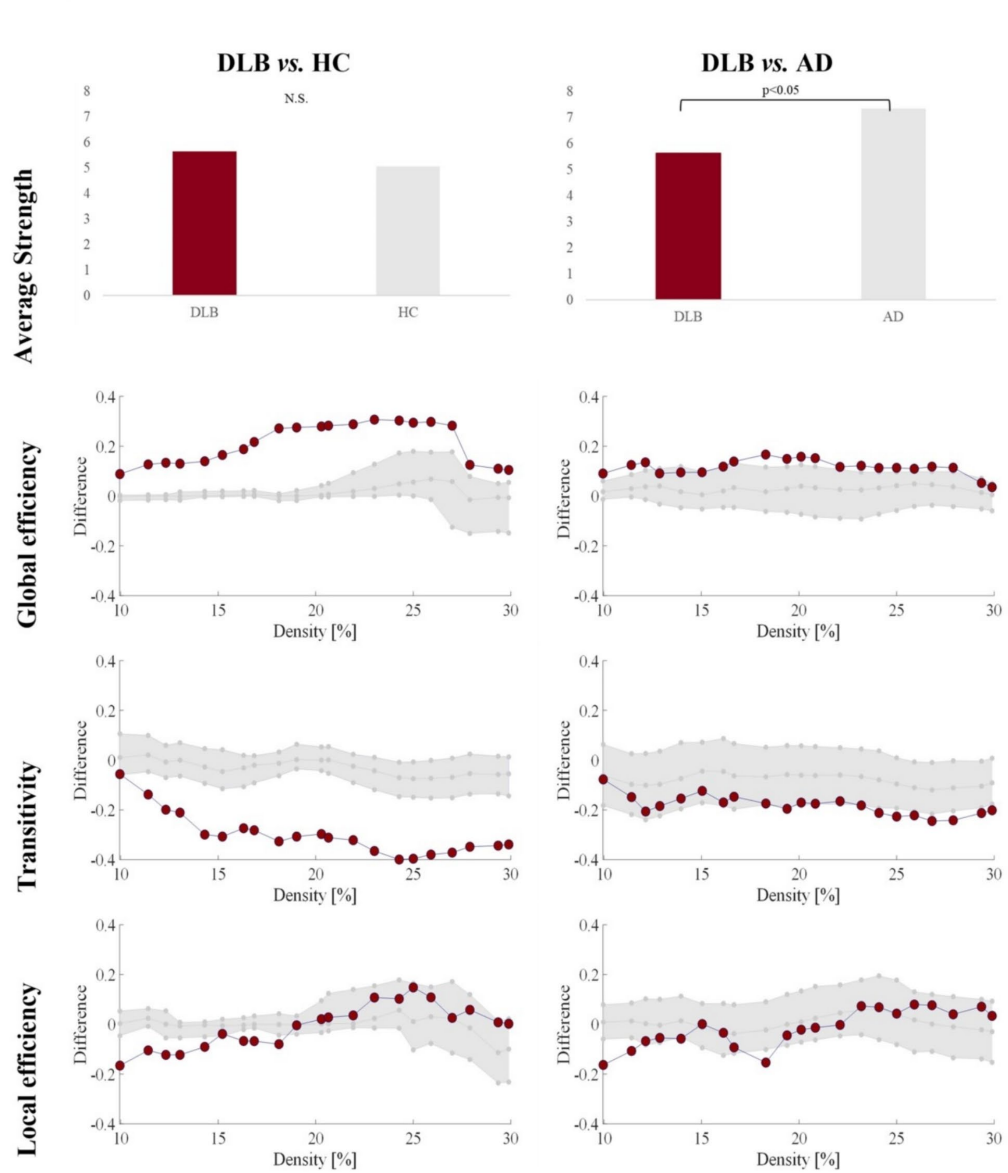
Differences of DLB patients with HC and AD groups in global graph measures. For global efficiency, transitivity, and local efficiency, connectome densities are displayed on the x-axis from min = 10% to max = 30%, in steps of 1%. Group differences are displayed on the y-axis. The grey area illustrates group differences with 95% confidence intervals. Red dots illustrate observed group differences. Negative differences indicate lower value in DLB compared to HC or AD, respectively. Positive differences indicate higher values in DLB compared to HC and AD, respectively. Between-group differences in global efficiency, transitivity, and local efficiency are significant when at least five consecutive red circles fall out of the grey-shaded area. Abbreviations: AD, Alzheimer’s disease; DLB, dementia with Lewy bodies; HC, healthy controls; N.S., non-significant.

Figure 2 shows quantitative differences in global graph measures. Compared with HC, DLB patients showed significantly higher global efficiency and a lower transitivity and local efficiency than HC, with no differences in the average strength. The differences for global efficiency and transitivity were more prominent than the differences for local efficiency. Compared with AD, DLB patients had a significantly lower average strength, higher global efficiency, and lower transitivity.

## Nodal graph measures

**Fig. 3:**
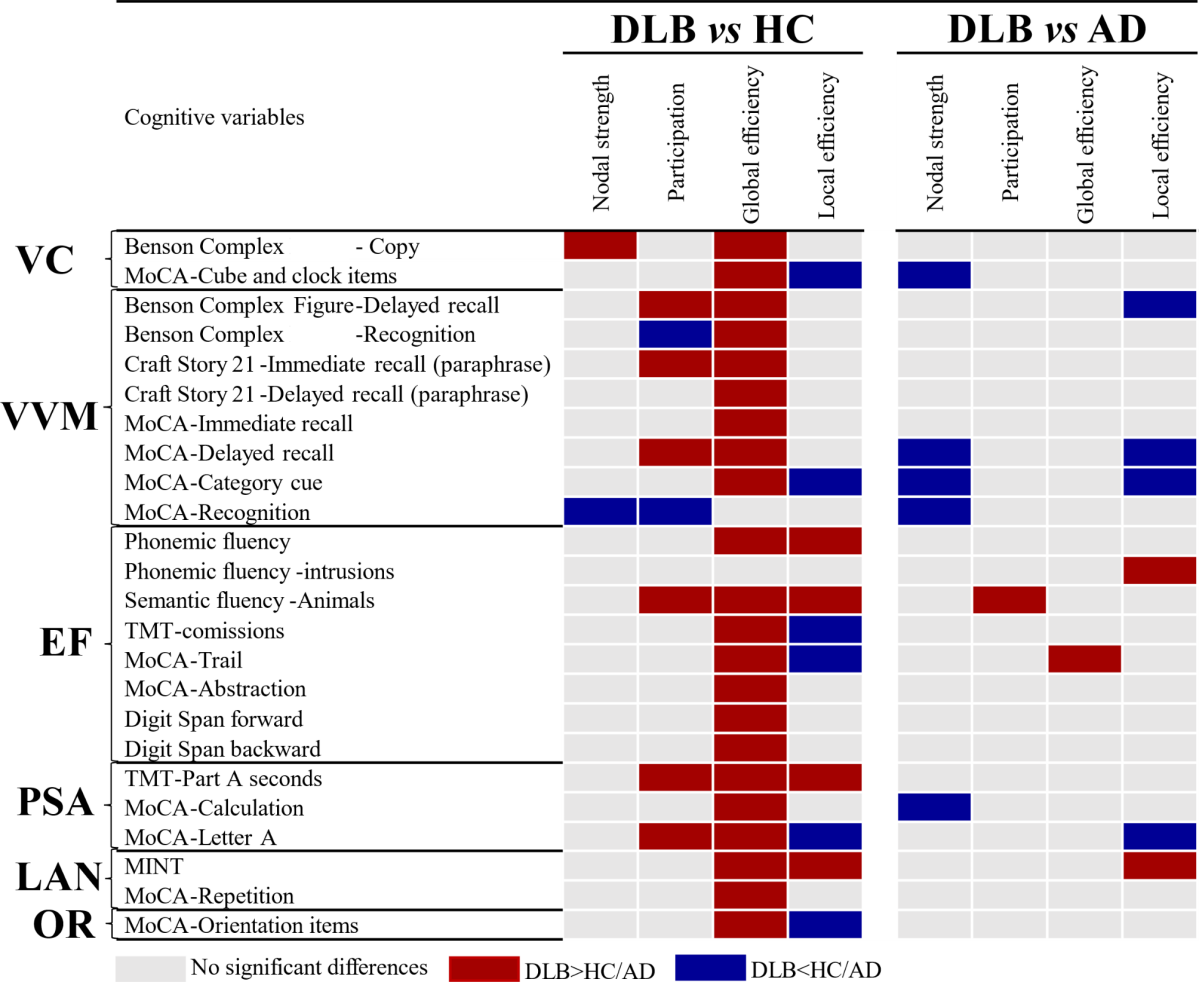
Summary of group differences in nodal graph measures. False discovery rate (FDR) adjustment at *p* ≤ 0.05 (two-tailed) in all comparisons. Abbreviations: VC, visuoconstructive functions; VVM, visual and verbal episodic memory; EF, executive functions; PSA, processing speed/attention; LAN, language; OR, orientation; TMT, Trail Making Test; MINT, Multilingual Naming Test. MoCA, Montreal Cognitive Assessment.

Figure 3 shows group differences in nodal measures. Overall, we observed more statistically significant differences in the comparison between DLB and HC than between DLB and AD.

When comparing DLB versus HC, global efficiency was the nodal measure that captured the most differences, indicating a higher nodal global efficiency in DLB in most cognitive measures except for the MoCA recognition measure and intrusions in phonemic fluency. Participation and local efficiency also captured several differences. Participation showed that free-recall memory, semantic fluency, and processing speed/attention had a higher participation in DLB than in HC. In contrast, recognition memory measures had a lower participation in DLB than in HC. The local efficiency measure showed generally lower values in DLB than in HC, involving all cognitive domains except language. We also observed higher values in local efficiency in DLB, particularly in executive, processing speed/attention, and language measures.

When comparing DLB versus AD, nodal strength and local efficiency were the measures that captured the most differences. Specifically, the DLB group showed a lower nodal strength in visuoconstructive, memory, and processing speed/attention measures compared with the AD group. Similarly, the DLB group showed a lower local efficiency in memory and processing speed/attention measures than the AD group. We also observed a higher local efficiency in DLB in intrusions of phonemic fluency and in naming in the language domain, as compared with AD.

## Discussion

We investigated the cognitive connectome of DLB patients using graph theory on cognitive measures and compared it with the cognitive connectome of HC individuals and AD patients. Our results showed alterations in the cognitive connectome of DLB patients driven by global and nodal alterations across features of integration, segregation, and centrality. These findings help characterize the complex associations among cognitive deficits in DLB patients, which can have clinical implications for differential diagnosis and cognitive interventions.

Our first objective was to characterize the cognitive connectome of DLB patients through a comparison with a HC group. Visual inspection of the connectomes showed that DLB patients had uniformly weak correlations within and between cognitive domains. In contrast, HC showed a functional small-worldness topology characterized by a balance between short and long connections. In our data, this was reflected by strong and weak correlations within and between cognitive domains. The pattern of correlations in DLB was substantiated by quantitative analyses of global and nodal graph measures. For global graph measures, quantitative analyses showed a higher global efficiency in DLB, which, in the face of lower cognitive performance compared with HC might reflect alterations in the connectome instead of a more efficient connectome in DLB. This is likely due to a loss of segregation in the connectome (as reflected by the lower transitivity and local efficiency in DLB). More specifically, the loss of segregation translates into cognitive nodes correlating with each other more sparsely, in the case of our DLB group, through weak correlations. Due to this widespread pattern of weak correlations, reaching any node in the connectome from any given node is easy, hence the higher global efficiency (and lower transitivity and lower local efficiency). However, this configuration is clearly aberrant, leading to the loss of a small-worldness topology in DLB. This combination of altered integration and segregation features has previously been reported in groups showing age-related cognitive decline^10,19^, and could be interpreted as a pattern of de-differentiation^20^. Indeed, the notion of de-differentiation refers to a higher intercorrelation between cognitive domains and has been associated with reduced neural specificity to cognitive processes^20–22^. Hence, our main finding of a cognitive de-differentiation suggests that the connectome of DLB patients has lost its cognitive specialization, i.e., cognition is no longer functionally organized in these patients, but rather, measures belonging to different cognitive domains start showing aberrant weak correlations with each other. Two previous studies also found a de-differentiation pattern in DLB using functional and metabolic imaging data^23,24^. Therefore, this de-differentiation finding may be consistent across different measures of neurodegeneration and downstream cognitive impairment in DLB. Future multimodal studies should confirm whether the cognitive de-differentiation observed in our study correlates with the de-differentiation of functional and metabolic brain networks.

Our nodal findings pinpointed connectome impairments in specific cognitive measures and domains. When comparing DLB with HC, the most prominent nodal findings emerged in the global efficiency measure, showing significantly higher values in DLB in all cognitive measures except for no statistically significant differences in one memory and one executive measure (i.e., MoCA-Recognition and phonemic fluency-intrusions). This abundance of differences in global efficiency in DLB reinforces the idea of a cognitive de-differentiation and is in line with the typical profile of cognitive impairment in DLB^25^. Deficits in recognition memory are not common in DLB. It is possible that recognition memory is not de-differentiated in DLB and therefore, recognition memory shows a pattern of correlations with other cognitive measures similar to that observed in HC. The absence of significant differences in phonemic fluency-intrusions may, however, be due to the low variability in that measure in our cohort.

Other less prominent nodal findings emerged in participation and local efficiency measures, when comparing DLB with HC. We observed a higher participation in DLB in free-recall memory, executive, and processing speed/attention measures. Higher participation exposes the cognitive measures with a more prominent role as connector hubs in a cognitive connectome. At the same time, hubs bear the risk of highly disrupting the connectome in case of failure^26^. The centrality of executive and processing speed/attention measures in our current study is in line with previous univariate DLB studies^4,27^, because cognitive measures that are more severely impaired tend to be more central in a cognitive connectome. These results are also consistent with neuroimaging studies showing alterations in attention networks in DLB^28^. Free-recall memory is a measure with an important executive component. Hence, the higher participation in free-recall memory could again be explained by the centrality of executive alterations in the connectome of DLB patients. In this line, a review suggested that deficits in free-recall in DLB may be secondary to the impairment in executive functions^7^. In contrast, we observed a lower participation in recognition memory in DLB. Recognition memory is more related to consolidation processes than to executive processes^29^. The lower participation of recognition memory may thus be explained by the known relative preservation of consolidation processes in DLB. Further, the fact that recognition memory was the only measure that showed differences in participation but not in global efficiency, suggests that centrality features compared to integration features might be more appropriate for investigating memory consolidation processes in DLB. For the results in nodal local efficiency, the predominance of lower values in DLB complements the results in global efficiency and supports the remarkable loss of segregation (de-differentiation). We only observed a higher local efficiency in some executive, processing speed/attention, and language measures, reminiscent of the typical profile of cognitive impairment observed in univariate studies in DLB^4,27^.

Our second objective was to investigate the cognitive connectome of DLB patients in comparison with that of AD patients, as the differential diagnosis of DLB with AD is common in the clinical setting. We observed less significant differences when comparing DLB with AD than with HC. Visual inspection of the cognitive connectomes showed a slightly more de-differentiated connectome in DLB than in AD, further characterized by several significant differences in quantitative analyses. Previous studies indicated that AD patients have alterations in integration and segregation features of their cognitive connectome^13,17,18^, whereas our direct comparison between DLB and AD suggests that those alterations might be more pronounced in DLB patients. Specifically, we found a lower segregation reflected by a lower transitivity, which coexisted with a higher global efficiency and a lower average strength in DLB than in AD. The lower average strength in DLB compared with AD reflects the generally weaker correlations in DLB and reinforces the idea that DLB shows a more de-differentiated cognitive connectome than AD. These cognitive findings may be primarily related to connectivity alterations and widespread hypometabolism, rather than atrophy or hypometabolism of a specific region^28^, as well as to the more prominent network alterations reported in DLB as compared with AD^30^. Hence, the findings in DLB seem more widespread or global than in AD, where the findings tend to be more regional or local, perhaps due to the central role of medial temporal neurodegeneration in AD^31^.

When comparing DLB with AD across nodal graph measures, the most prominent findings emerged in nodal strength and local efficiency, indicating a lower centrality and segregation in DLB. The reduced nodal strength in DLB included measures related to recall and consolidation in verbal memory, visuoconstruction, and calculation. Our results for memory are in line with previous studies using graph theory on cognitive data that reported the role of memory measures in the connectome of AD patients^13,17,18^. The lower nodal strength in visuoconstruction and calculation measures in DLB is in line with previous univariate studies showing a lower performance in visual abilities and attention in DLB than in AD^32^. Regarding the nodal local efficiency, DLB patients had a lower local efficiency than AD patients particularly in memory measures. Similar to our interpretations for the nodal global efficiency, this finding in local efficiency may reflect the typically better performance in memory in DLB than in AD^32^. Overall, these nodal results offer a multivariate understanding of the specific cognitive measures that may best help discriminate DLB from AD, once considering the complex associations and interactions among multiple cognitive impairments.

This study extends the findings from previous univariate studies [25 for a review] by demonstrating that DLB patients not only present deficits in specific cognitive functions such as attention, executive functions and visual abilities, but they also present with overt alterations in their cognitive connectome. This finding may have clinical implications. Firstly, advancing our currently limited understanding of the complex associations among cognitive measures and clinical phenotype in DLB may help improve its differential diagnosis with AD and other dementias. Particularly, our nodal analyses offer some insights into cognitive measures that can guide or assist in such differential diagnosis. An interesting prospect for future studies would be to test the ability of the cognitive connectome to discriminate DLB from HC and AD, for example using machine learning in a classification task. It would also be interesting to expand our current analyses towards comparing the cognitive connectome of DLB patients with the connectome of patients with Parkinson’s Disease with Dementia, who share clinical and pathological similarities with DLB. Secondly, characterizing the cognitive connectome of DLB patients can help predict the compensation of cognitive deficits in a specific node and identify targets for cognitive stimulation^16^. For example, our findings suggest that DLB patients could benefit from interventions against memory impairment that target compensation strategies (category cue or recognition). In contrast, AD patients could benefit from interventions underpinning consolidation processes. The connectome data suggest what other cognitive processes could be leveraged to compensate for or stimulate memory differentially in DLB and AD. This means that the knowledge about the cognitive connectome helps understand how interventions in a given node might promote transferred effects to other cognitive nodes.

This study has some limitations. The availability of data was not even across cognitive measures, which may have led to the underrepresentation of some cognitive domains. To mitigate this, we focused our interpretations and conclusions on cognitive domains or processes rather than focusing on specific cognitive measures. Furthermore, the properties of a connectome can change with the addition or removal of a node^33^. To address this, we included as many nodes as possible, without disregarding nodes that are relevant for understanding cognitive functioning in pathological populations, such as error measures. Another limitation is that our connectome included several cognitive measures from the same test (i.e. MoCA). While the constructed connectome allowed us to capture significant differences between DLB patients and controls and AD patients, future studies should extend our analyses towards including more comprehensive cognitive assessments. Finally, diagnosis of DLB was entirely clinical, while the underlying Lewy body-related pathology can only be confirmed post-mortem. While the NACC dataset provides neuropathological confirmation for some cases, such information was available for too few DLB cases, precluding us to perform sensitivity analyses.

In conclusion, we demonstrated that the cognitive connectome of DLB patients is altered. This alteration reflects a severe loss of segregation leading to a pattern of cognitive de-differentiation, with the disease particularly targeting processing speed/attention, executive functions, and free-recall memory. Hence, the connectome of DLB patients has lost its cognitive specialization. The comparison with AD patients showed the specificity of the DLB findings and uncovered the role of free-recall memory in DLB in contrast to consolidation of memory in AD. This study helps advance our current understanding of cognitive impairment and clinical phenotype in DLB, and aids in its clinical discrimination from AD, a diagnostic group that can be confused with DLB clinically.

### Methods

#### Participants

We obtained data from the National Alzheimer’s Coordinating Center (NACC, National Institute on Aging at the National Institutes of Health (NIA/NIH) Grant U24-AG072122)^34^, collected across 32 AD Research Centers between March 2015 and May 2021. Patients aged ≥ 45 years and diagnosed with DLB or AD according to the McKeith criteria for DLB and National Institute on Aging and Alzheimer’s Association (NIA-AA) criteria for AD^2,31^ were included in the current study. Clinical severity was assessed using the Clinical Dementia Rating (CDR) scale^35^. Core clinical features of DLB were determined by the clinician’s judgment, including fluctuating cognition, visual hallucinations, probable rapid eye movement sleep behavior disorder (RBD), and parkinsonism. We also included a group of HC who demonstrated unimpaired cognition during the clinical assessment, available in the NACC dataset as well.

All participants were required to have data available on all the cognitive measures selected for the construction of the cognitive connectomes (see next section). For all participants, the exclusion criteria were having a clinical history of bipolar disorder, schizophrenia, delusional disorder, craniocerebral trauma, stroke, substance abuse, uncorrected vision, or hearing problems. Written informed consent was obtained at individual AD Research Centers and approved by local Institutional Review Boards.

## Cognitive measures and construction of the cognitive connectome

The neuropsychological protocol of the NACC database is fully described elsewhere^34^. We included 25 cognitive measures that mapped multiple cognitive domains, such as visuoconstructive functions, visual and verbal episodic memory, executive functions, processing speed, attention, language, or orientation. Variables with missing values in more than 15% of the DLB patients such as the Trail Making Test-Part B were excluded. Additionally, for the univariate characterization of our study groups and interpretation of correlation matrices, we grouped the included cognitive measures into six cognitive domains based on standard classifications from previous studies (see Table 2)^10,36,37^.

Before constructing the cognitive connectome, we carefully inspected the distribution and nature of all 25 cognitive measures. We inverted the scores when necessary, so that higher scores always indicated a better performance, and transformed skewed measures to facilitate subsequent statistical analyses. Moreover, since age, sex, and education are associated to cognitive performance, we removed their effects on all cognitive measures using multiple linear or logistic regressions as detailed in Amato and colleagues^38^. After these steps, data was inspected again to ensure that all measures in the new dataset were normally distributed. This inspection demonstrated that one measure did not follow the normal distribution. Therefore, we chose Spearman correlation coefficients to define the edges of the cognitive connectomes. At that point, we observed that one measure (i.e., repetition errors in phonemic fluency) was barely correlated with the rest of the measures and was thus excluded for further statistical analysis since we aimed to investigate fully connected cognitive connectomes. The remaining 24 cognitive measures were used to construct a cognitive connectome for each study group, i.e., DLB, AD, and HC (Table 2).

We constructed correlation matrices using positive and negative correlations, as in previous studies on cognitive connectomes^15,16^. Self-connections were excluded from the correlation matrices. Next, correlation matrices were binarized by thresholding coefficients at a range of densities (fraction of connected edges), defined based on the cognitive connectome of the HC group. Initial analysis of the cognitive connectome of HC showed that MoCA variables tended to form a cluster of correlations, increasing the threshold density at which other nodes became connected. We thus built a simplified connectome without MoCA variables to determine the range of densities in HC. At the minimum density (10%), nodes tended to be connected to at least one other node. At the maximum density (30%), the connectome exhibited a random topology with the small-world index reaching < 1. We returned to the full connectomes, including MoCA variables, and inspected the suitability of a 10-30% range of densities in all three study groups. The suitability of the 10-30% range was confirmed by ensuring that there were generally no disconnected connectomes or random topologies. We accepted that range of densities, while our inspection demonstrated that particularly the connectome of DLB patients tended to exhibit a random topology with the small-world index reaching < 1 before the density of 30%.

**Table 2:** List of cognitive measures included in the cognitive connectomes.

Cognitive measures	Cognitive domains
Benson Complex Figure-Copy	Visuoconstructive functions
MoCA-Cube and clock items	
Benson Complex Figure-Delayed recall	Visual and verbal episodic memory
Benson Complex Figure-Recognition	
Craft Story 21-Immediate recall (paraphrase)	
Craft Story 21-Delayed recall (paraphrase)	
MoCA-Immediate recall	
MoCA-Delayed recall	
MoCA-Category cue	
MoCA-Recognition	
Verbal fluency-Phonemic Verbal fluency-Phonemic-intrusions Verbal fluency-Semantic (Animals)	Executive functions
Trail Making Test-commissions	
MoCA-Trail	
MoCA-Abstraction	
Digit span forward Digit span backward	
TMT-Part A seconds	Processing speed/attention
MoCA-Calculation	
MoCA-Letter A	
Multilingual Naming Test	Language
MoCA-Repetition	
MoCA-Orientation items	Orientation

For most cognitive measures in the table, performance reflects the number of correct elements otherwise specified in the table (i.e., intrusions, commissions, and seconds). Abbreviations: MoCA, Montreal Cognitive Assessment; TMT, Trail Making Test.

### Graph theory measures

To investigate the DLB cognitive connectome, we calculated graph measures that reflect centrality, integration, and segregation features at global and nodal levels. Among the different graph measures available, we selected those that demonstrated stability in previous studies^39^ and had been used to investigate cognitive connectomes^10^. Specifically, we calculated the global measures of average strength (a measure of the magnitude of correlations), global efficiency (a measure of integration), transitivity (a measure of segregation), and local efficiency (a measure of segregation)^40^. We also calculated the nodal measures of strength (a measure of centrality) and participation (a measure of centrality), global efficiency, and local efficiency^40^. All graph measures are fully described in Supplementary Table 1. We calculated all graph measures on binary networks except for global and nodal strength, which were calculated on the weighted network (before binarization).

### Statistical analysis

We used ANOVA for between-group comparisons of demographic and clinical variables and ANCOVA with age, sex, and education as covariates for between-group comparisons of the six cognitive domains. The six cognitive domains were obtained by computing z-scores of the original 24 cognitive measures using means and standard deviations of the HC group as the reference; and then averaging those z-scores into domains as detailed in Table 2. An exception was made for orientation, which consisted only of one z-score. Statistical significance was set at *p* < 0.05 in all these analyses.

Between-group comparisons of graph measures were conducted through 1000 nonparametric permutations. Results from global graph measures were reported across connectome densities from 10 to 30%, in steps of 1%. We report and interpret global measures that were significant in ≥ 5 consecutive densities to focus on stable differences across the range of densities^41^. For nodal measures, the false discovery rate (FDR) adjustment for multiple testing was used at *p* ≤ 0.05 (two-tailed)^42^. We report nodal results at the median density (20%), as in previous studies^10,43^. Nonetheless, we also ensured that results at the median density were representative of neighbouring densities to focus on stable differences across the range of densities.

Statistical analyses were performed using R Studio with the ULLRToolbox, SPSS version 25.0, and BRAPH software version 1.0.0^44^.

## Electronic supplementary material

Below is the link to the electronic supplementary material.

Supplementary Material 1

## Data Availability

The data used in this study is from the NACC. Data was available to the authors through https://naccdata.org/. For further information upon data availability contact DF.
