# Supplementary material for: Patients with dementia with Lewy bodies display a signature alteration of their cognitive connectome
Source: Sci Rep. 2025 Jan 6;15:940. doi: 10.1038/s41598-024-84946-4 (PMC11704352; doi:10.1038/s41598-024-84946-4)
Supplement: Supplementary file 1 — Supplementary Material 1 [file 41598_2024_84946_MOESM1_ESM.docx]

**SUPPLEMENTARY MATERIALS**

**Patients with dementia with Lewy bodies display a signature alteration of their cognitive connectome**

Roraima Yanez-Perez^1,2^, Eloy Garcia-Cabello^3^, Annegret Habich^1,4^, Nira Cedres^1,3,5^, Patricia Diaz-Galvan^6,7^, Carla Abdelnour^8^, Jon B. Toledo^9^, José Barroso^3^ and Daniel Ferreira^1,3,6^

^1^ Division of Clinical Geriatrics, Centre for Alzheimer Research, Department of Neurobiology, Care Sciences, and Society, Karolinska Institutet, Stockholm, Sweden

^2^ Department of Clinical Psychology, Psychobiology and Methodology, Faculty of Psychology, University of La Laguna, Canary Islands, Spain

^3^ Department of Psychology, Faculty of Health Sciences, University Fernando Pessoa Canarias, Las Palmas, Spain.

^4^ University Hospital of Psychiatry and Psychotherapy, University of Bern, Bern, Switzerland

^5^ Department of Psychology, Sensory Cognitive Interaction Laboratory (SCI-lab), Stockholm University, Stockholm, Sweden

^6^ Department of Radiology, Mayo Clinic, Rochester, Minnesota, United States

^7^ Unidad de Trastornos del Movimiento, Servicio de Neurología y Neurofisiología Clínica, Instituto de Biomedicina de Sevilla, Hospital Universitario Virgen del Rocío/CSIC/Universidad de Sevilla, Seville, Spain

^8^ Department of Neurology and Neurological Sciences, Stanford University School of Medicine, Stanford, California, United States

^9^ Nantz National Alzheimer Center, Stanley H. Appel Department of Neurology, Houston Methodist Hospital, Houston, Texas, United States

**Table S1** Graph Theory Measures

| Measures | | Definition |
| --- | --- | --- |
| Global efficiency | Nodal global efficiency [1] | Average of the inverse shortest path length between a specific node and the rest of the network. |
|  | Average global efficiency [1] | Average of the global efficiencies of all nodes. It measures how efficiently information is exchanged throughout the network. This measure, in contrast to the characteristic path length, can be computed on disconnected networks [2] |
| Local efficiency | Nodal local efficiency [1] | Global efficiency of a node calculated on the subgraph created by the node’s neighbors. |
|  | Average local efficiency [1] | Average of the local efficiencies of all nodes. |
| Strength | Nodal strength [3] | Sum of the weights of all edges connected to a node. |
|  | Average strength [4] | Average of the strengths of all nodes. |
| Transitivity [4] | | Fraction of a node’s neighbors that are also neighbors of each other in the whole network, normalized by the whole network, reflecting how well the nodes are connected to nearby regions forming cliques. The transitivity measure is similar to the clustering coefficient but is less vulnerable to methodological issues (e.g., edge definition, network size, and groups composition) [5, 6] |
| Participation [7] | | Quantifies the relation between the number of edges connecting a node outside its community and its total number of edges. |

**REFERENCES SUPPLEMENTARY MATERIALS**

1. Latora, V. & Marchiori, M. Efficient Behavior of Small-World Networks. *Phys. Rev. Lett.* **87**, 198701 (2001).

2. Ferreira, D., Pereira, J. B., Volpe, G. & Westman, E. Subtypes of Alzheimer’s Disease Display Distinct Network Abnormalities Extending Beyond Their Pattern of Brain Atrophy. *Front. Neurol.* **10**, 524 (2019).

3. Barrat, A., Barthélemy, M., Pastor-Satorras, R. & Vespignani, A. The architecture of complex weighted networks. *Proc. Natl. Acad. Sci. U.S.A.* **101**, 3747–3752 (2004).

4. Rubinov, M. & Sporns, O. Complex network measures of brain connectivity: Uses and interpretations. *NeuroImage* **52**, 1059–1069 (2010).

5. Mårtensson, G. *et al.* Stability of graph theoretical measures in structural brain networks in Alzheimer’s disease. *Sci Rep* **8**, 11592 (2018).

6. Phillips, D. J., McGlaughlin, A., Ruth, D., Jager, L. R. & Soldan, A. Graph theoretic analysis of structural connectivity across the spectrum of Alzheimer’s disease: The importance of graph creation methods. *NeuroImage: Clinical* **7**, 377–390 (2015).

7. Mijalkov, M. *et al.* BRAPH: A graph theory software for the analysis of brain connectivity. *PLOS ONE* **12**, e0178798 (2017).
